# Physico-Chemical and Structural Interpretation of Discrete Derivative Indices on N-Tuples Atoms

**DOI:** 10.3390/ijms17060812

**Published:** 2016-05-27

**Authors:** Oscar Martínez-Santiago, Yovani Marrero-Ponce, Stephen J. Barigye, Huong Le Thi Thu, F. Javier Torres, Cesar H. Zambrano, Jorge L. Muñiz Olite, Maykel Cruz-Monteagudo, Ricardo Vivas-Reyes, Liliana Vázquez Infante, Luis M. Artiles Martínez

**Affiliations:** 1 Computer-Aided Molecular “Biosilico” Discovery and Bioinformatic Research International Network (CAMD-BIR IN), Cumbayá-Tumbaco, Quito 170184, Ecuador; oscarms@uclv.edu.cu (O.M.-S.); sjbarigye@yahoo.com (S.J.B.); dirfabrica@eppc.cfg.minag.cu (L.V.I.); jmuniz@unitecnologica.edu.co (L.M.A.M.); 2 Department of Chemical Science, Faculty of Chemistry-Pharmacy, Universidad Central “Martha Abreu” de Las Villas, Santa Clara 54830, Villa Clara, Cuba; 3 Universidad San Francisco de Quito (USFQ), Grupo de Medicina Molecular y Traslacional (MeM&T), Colegio de Ciencias de la Salud (COCSA), Escuela de Medicina, Edificio de Especialidades Médicas, Hospital de los Valles, Av. Interoceánica Km 12 ½—Cumbayá, Quito 170157, Ecuador; 4 Universidad San Francisco de Quito (USFQ), Instituto de Simulación Computacional (ISC-USFQ), Diego de Robles y vía Interoceánica, Quito 170157, Ecuador; jtorres@usfq.edu.ec (F.J.T.); czambrano@usfq.edu.ec (C.H.Z.); 5 Grupo de Investigación Microbiología y Ambiente (GIMA), Programa de Bacteriología, Facultad Ciencias de la Salud, Universidad de San Buenaventura, Calle Real de Ternera, Cartagena de Indias, Bolívar 130010, Colombia; 6 Departamento de Química, Universidade Federal de Lavras (UFLA), Caixa Postal 3037, Lavras 37200-000, MG, Brazil; 7 School of Medicine and Pharmacy, Vietnam National University, Hanoi (VNU) 144 Xuan Thuy, Cau Giay, Hanoi 100000, Vietnam; ltthuong1017@gmail.com; 8 Universidad San Francisco de Quito (USFQ), Grupo de Química Computacional y Teórica (QCT-USFQ), Departamento de Ingeniería Química, Diego de Robles y Vía Interoceánica, Quito 170157, Ecuador; 9 Grupo de Investigación en Estudios Químicos y Biológicos, Facultad de Ciencias Básicas, Universidad Tecnológica de Bolívar (UTB), Parque Industrial y Tecnológico Carlos Vélez Pombo Km 1 vía Turbaco, Cartagena de Indias, Bolívar 130010, Colombia; jmuniz@unitecnologica.edu.co; 10 Instituto de Investigaciones Biomédicas (IIB), Universidad de Las Américas (UDLA), Quito 170513, Ecuador; maykel.cruz@udla.edu.ec; 11 Grupo de Química Cuántica y Teórica, Facultad de Ciencias, Universidad de Cartagena, Cartagena de Indias, Bolívar 130001, Colombia; rvivasr@unicartagena.edu.co; 12 Grupo CipTec, Fundación Universitaria Tecnológico de Comfenalco, Facultad de Ingenierías, Programa de Ingeniería de Procesos, Cartagena de Indias, Bolívar 130001, Colombia

**Keywords:** discrete derivative, GDIs, derivative indices, structural interpretation, reactivity, activation entropy, ^17^O-RMN, free valence, resonance energy

## Abstract

This report examines the interpretation of the Graph Derivative Indices (GDIs) from three different perspectives (*i.e.*, in structural, steric and electronic terms). It is found that the individual vertex frequencies may be expressed in terms of the geometrical and electronic reactivity of the atoms and bonds, respectively. On the other hand, it is demonstrated that the GDIs are sensitive to progressive structural modifications in terms of: size, ramifications, electronic richness, conjugation effects and molecular symmetry. Moreover, it is observed that the GDIs quantify the interaction capacity among molecules and codify information on the activation entropy. A structure property relationship study reveals that there exists a direct correspondence between the individual frequencies of atoms and Hückel’s Free Valence, as well as between the atomic GDIs and the chemical shift in NMR, which collectively validates the theory that these indices codify steric and electronic information of the atoms in a molecule. Taking in consideration the regularity and coherence found in experiments performed with the GDIs, it is possible to say that GDIs possess plausible interpretation in structural and physicochemical terms.

## 1. Introduction

Many mathematical invariants used in the codification of chemical information of molecular structures have in the recent years gained important utility in several research fields [1,2,3]. These invariants are more advantageous relative to physicochemical parameters customarily employed in describing, for instance, the hydrophobic, steric and/or electronic effects due modifications of substituents in a molecule (e.g., the Hammett’s sigma constant); because they are able to quantify greater chemical information on molecules and usually yield better performance in studies on structure-property/activity relationships, similarity/diversity, virtual screening, among others. These aforementioned mathematical invariants are known as Molecular Descriptors (MDs).

Formally, MDs may be defined as the final result of a logical and mathematical procedure in which the chemical information codified in a symbolic representation of the molecule is transformed into a number [4].

When MDs are based on the topology of the molecular structure, these are denominated as Topological Indices (TIs), which are derived from a graph-theoretical invariant and are able to codify information on molecular connectivity [5]. In other words, the TIs are numeric representations of the molecular structure and should encode useful structural characteristics such as: size, symmetry, ramifications, cycles, type of atoms, as well as the multiplicity of bonds in a chemical structure.

One of the 13 properties proposed by Randic as desirable for any MD is the direct structural interpretation [6]. Additionally, the fifth aspect taken into account to validate a Quantitative Structure-Active Relationship QSAR model [7] is related with its interpretation, which is directly related to the understanding of MDs in structural and/or physicochemical terms. However, the majority of the research efforts have been focused on applying these MDs (TIs) in a significant number of *in silico* molecular modeling tasks and the virtual screening of chemical compounds of interest. Efforts destined to the direct interpretation of the majority of the graph-theoretical invariants in structural and/or physicochemical terms are generally rare [8]. This fact is clearly portrayed in statement by Hoffmann that [9], “In many interesting areas of chemistry we are approaching predictability, but I would claim, not understanding”.

MDs will probably play an increasing role in computational, theoretical and medicinal chemistry. In fact, the availability of a large number of theoretical MDs containing diverse sources of chemical information would be useful to comprehend better the relationship between molecular structures and experimental evidence, taking advantage of the increasingly more powerful methods, computational algorithms and fast computers [4].

The previous affirmation demonstrates the increasing need to find some nexus among modern TIs with familiar concepts in established sciences, in order to optimize known procedures, group similar methods and create new MDs that codify orthogonal chemical information, and ultimately enhance their utility and scope of application.

Recently, some of the authors of the present work defined a new family of TIs based on the concept of the discrete derivative (specifically, derivative of a molecular graph), which was denominated Graph Derivative Indices (GDI) [10,11]. These GDIs have been applied in several Quantitative Structure/Property Relationship (QSAR/QSPR) studies showing satisfactory results [10,11,12]. These indices have been defined as global and local invariants for atoms and/or group of atoms and are based on the concept of discrete derivative of a Graph “G” with respect to an event “S” over duplex, triplex and quadruplex relations of atoms (vertexes) [10,12].

The concept of the graph-based derivative with respect to a given event “S” was proposed initially by V. A. Gorbátov in 1988 [13]. To evaluate the derivative of a group of elements belonging to a graph it is necessary to know the individual and the reciprocal participation frequencies of the graph elements (vertexes), in the set of conditions (sub-graphs), for which the event is true [10,11,13]. The derivative values characterize the non-homogeneous participation degree of the groups of elements from a graph in a given event [10,13].

The derivative value over *n*-elements of a G can be obtained as:

(1) $$\frac{\partial G}{\partial S}(m_{1},m_{2},...m_{n})=\frac{1}{f_{m_{1},m_{2},...m_{n}}}\left(\sum_{i}f_{i}-2\cdot \sum_{\begin{array}{l} i_{1},i_{2}\\ i_{1}\neq i_{2} \end{array}}f_{i_{1}i_{2}}+...+{\left(-1\right)}^{\alpha +1}\cdot \alpha \cdot \sum_{\begin{array}{c} i_{1},i_{2}...i_{\alpha }\\ i_{1}\neq i_{2},...,i_{\alpha -1}\neq i_{\alpha } \end{array}}f_{i_{1}i_{2}...i_{\alpha }}+...+{\left(-1\right)}^{n+1}\cdot n\cdot \sum_{\begin{array}{c} i_{1},i_{2},...,i_{n}\\ i_{1}\neq i_{2},...,i_{n-1}\neq i_{n} \end{array}}f_{i_{1}i_{2}...i_{n}}\right)$$

If we wish to know the derivative over a pair (*i,j*) of elements from a graph (duplex), the Equation (1) may be simplified to:

(2) $$\frac{\partial G}{\partial S}\left(i\;j\right)=\frac{f_{i}-2f_{ij}+f_{j}}{f_{ij}}$$

The Equation (1) (and Equation (2)) is the main expression for the underlying concept of the GDIs, thus a reliable interpretation of its elements is necessary to get an adequate understanding of the indices in terms of physical observables associated to the molecular structure.

In this report, a deduction of the graph discrete derivative, analogous with the classical derivative from mathematical analysis will be presented. This analysis will be used as basis for getting physicochemical and structural interpretation of the derivative values over atom-pairs. Besides, a group of carefully designed experiments to corroborate the veracity of the propositions made in the GDIs interpretation will be reported.

## 2. Graph Derivative Indices

### 2.1. Discrete Derivative. Deduction and Analogy with the Classical Derivative Concept

Isaac Newton is credited with the pioneer development of modern-day differential calculus, built on earlier work by Fermat, Barrow and Descartes. He defined the derivative of the function y=f(x) at the point *x*, as the limit of the differences relation as Δx→0:

(3) $$\frac{\text{dy}}{\text{dx}}=\underset{\Delta x\to o}{\lim}\frac{\text{Δy}}{\text{Δx}}=\underset{\Delta x\to 0}{\lim}\frac{f\left(x+\Delta x\right)-f\left(x\right)}{\Delta x}$$

**Figure 1:**
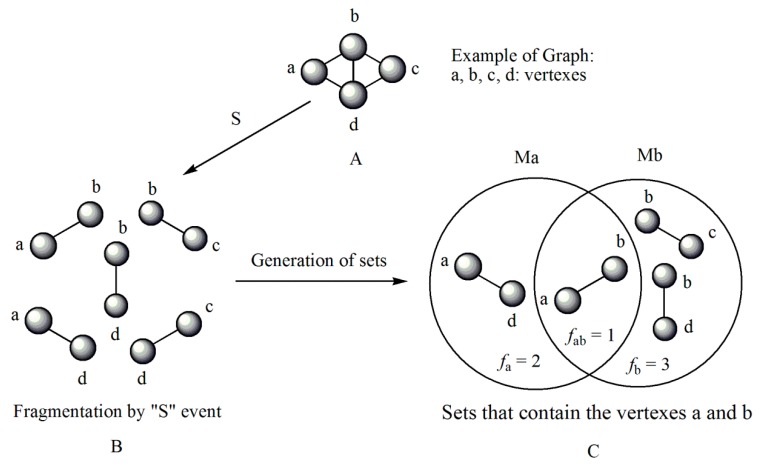
Example of graph G (**A**), fragmentation according to an event “S” (**B**) and the sub-graphs sets for vertexes a and b (**C**).

Note that this notation was proposed by Leibnitz [14]. In the mathematical analysis the derivative characterizes a function’s variation degree when a small variation in its argument is made, that is, the classical definition of the derivative is based on the concept of the limit [15]. In discrete mathematics the definition of the limit does not hold due to the non-continuous nature of the functions, therefore it is impossible to establish an exact application of the derivative concept from continuous to discrete mathematics [13]. However to solve optimization problems in discrete mathematics, the discrete derivatives is introduced based on the use of the frequency of letters in words of a *ψ* model. In order to illustrate this concept, a graph G composed of four vertexes and five edges, as shown in Figure 1, is taken as an example. A new event “S” is defined (Figure 1B) so that it is possible to obtain fragments of G in “5” sub-graphs (words from model-collection of conditions for which the event is true).

It is feasible to characterize the participation frequency of conditions (letters, atoms) in the collections of conditions (words, sub-structures) in which the event is true using the corresponding set of conditions. For vertexes “a” and “b” the corresponding frequencies would be *f*_a_ = 2, *f*_b_ = 3. With this scheme is also possible to determine the simultaneous inclusion’s frequency for vertexes “a” and “b”, *i.e.*, the Reciprocal Frequency (*f*_ab_). For the example from Figure 1, the *f*_ab_ = 1. Based on latter analysis, we may conclude that for any pair of vertexes (letters) *i* & *j* from a G, the individual frequencies are greater or equal to the reciprocal frequencies between them, that is $f_{i}\geq f_{ij}$ and $f_{j}\geq f_{ij}$ [10,11,13].

Up to now two sub-graph sets are defined, namely M_a_ and M_b_, obtained by graph fragmentation, taking as base the application of a given event. Both sets may or may not have coincident elements (sub-graphs that contain “a” and “b” vertexes at the same time). The number of coincident sub-graphs is quantified by the reciprocal frequency of vertexes “a” and “b” (*f*_ab_) and expresses the magnitude of the separation of these sets. Therefore, *f*_ab_ is similar to the increase or perturbation ∆*x*, defined in classical mathematics.

Maintaining the analogy with mathematical analysis, it is possible to evaluate de variation’s degree as the symmetrical difference between sets, as shown in Equation 4:

(4) $$M_{a}\Delta M_{b}=\left(M_{a}\backslash M_{b}\right)\cup \left(M_{b}\backslash M_{a}\right)$$

If this expression is written taking as base the individual and reciprocal frequencies, it would be:

(5) $$M_{a}\Delta M_{b}=\left(f_{a}-f_{ab}\right)+(f_{b}-f_{ab})$$

(6) $$M_{a}\Delta M_{b}=f_{a}-2f_{ab}+f_{b}$$

Dividing Equation (6) by *f*_ab_, which is representative of the perturbation degree from a set to another, and expressed in terms of the differentiation with respect to the event, Equation (7) is obtained:

(7) $$\frac{dG}{dS}\left(a\;b\right)=\frac{f_{a}-2f_{ab}+f_{b}}{f_{ab}}$$

The mathematical Expression (7) is denominated as the graph derivative with respect to an event over a pair of vertexes (duplex relations), and it defines the non-uniform participation degree in the “S” event of the pair (a,b) belonging to G. In other words, Equation (7) expresses the heterogeneity degree of pairs of components constituting G with respect to any previous event [10,11,13] and it can be interpreted as a non-directed weighed graph <V,(U,P)> whose bearer corresponds with that of a model determined by this event and vertexes (v*_i_*, v*_j_*), weighted by the ratio of the incompatible frequency [(*f_i_ − f_ij_*) + (*f_j_ − f_ij_*)] with the compatible frequency *f_ij_* in the event, and with a particularity that [13]:

$$\left(v_{i},v_{j}\right)\notin U,\text{ if }\frac{\text{dG}}{\text{dS}}\left(v_{i},v_{j}\right)=\;\infty$$

$$\left(v_{i},v_{j}\right)\in U,\text{ if }\frac{\text{dG}}{\text{dS}}\left(v_{i},v_{j}\right)=\text{finite magnitude different of zero}$$

$$\left(v_{i}=v_{j}\right),\text{ if }\frac{\text{dG}}{\text{dS}}\left(v_{i},v_{j}\right)=0$$

**Figure 2:**
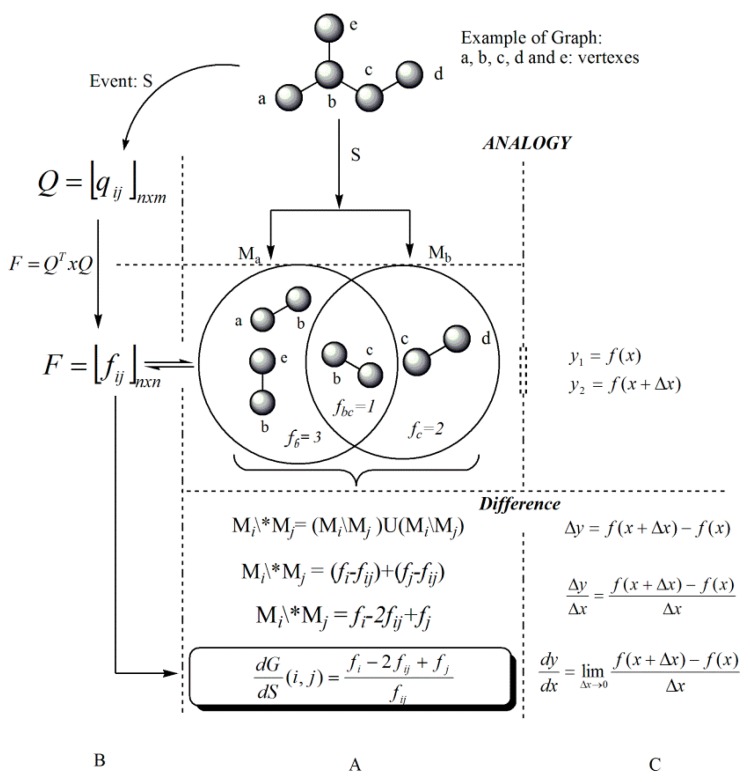
Scheme of the analogy between derivatives and their mathematical development. (**A**) Obtaining of the discrete derivative over a pair of elements *i j* from a graph G; (**B**) Algebraic development of the process for obtaining the discrete derivative over pairs of vertexes; (**C**) Obtaining of the classical derivative from the mathematical analysis.

A scheme that summarizes the analogy between discrete and continuous derivatives is shown in Figure 2. Note that various events may be applied to a given model; thus allowing for varied information to be retrieved from the chemical structure, and ultimately yielding different MDs [16]. Recently 12 theoretical events were introduced and these include: connected sub-graphs (S), walks of length k (K), Sach’s sub-graph (H), Quantum (Q), Terminal Path (T), path-vertex incidence (V), Multiplicity (M), MACC fingerprint (C), Sub-structures fingerprint (B), E-State fingerprint (E), Alog P (A), and Refractivity (R). The application of these events has allowed for the obtaining of varied information and generating a high number of MDs that characterize the chemical structure from dissimilar perspectives. These events have been successfully applied in several applications and are grouped in three clusters: Topological events (S, K, H, Q, T, V and M), Fingerprint-based events (C, B and E) and Physico-chemical events (A and R) [3,16].

It is also important to analyze the characteristics of the Relations Frequency Matrix (F), given that its elements make possible the evaluation of the discrete derivatives. The matrix F is an *n × n* symmetrical matrix (where, *n* is the number of atoms in the molecule), whose diagonal elements are denominated as Individual Frequencies (*f_i_*) of each element in G, and the off-diagonal elements correspond to the Reciprocal Frequencies.

To determine the derivatives over *n*-elements as shown in the Equation (1), F would be an *n*-dimensional matrix (or so-called hypermatrix). In a previous report, the GDIs have been generalized for calculating derivatives over *n*-tuples of atoms [12], and a similar theoretical scaffold was also applied in generalizing the GT-STAF (acronym for Graph Theoretical Thermodynamic STAte Functions) indices based on information theory [17], From this point onwards the attention will be focused on the graph derivative over duplex relations, which will be calculated when the molecule is fragmented according to an event criteria S and using order 1 substructures. This easy description of the molecular structure allows arriving at conclusions which will serve as a base for the interpretation of the values of the discrete derivative over a pair of atoms and posteriorly generalized to *n*-tuples of atoms.

### 2.2. Graph Derivative Indices (GDI). Application to Chemical Codification

The main goal of this manuscript is to find the structural and/or physicochemical interpretation of GDIs. Although GDIs have been successfully defined and applied in several studies [10], it is necessary to remember some medullar aspects of their theory and as well as the corresponding mathematical algorithms used in the description of the organic structure.

**Figure 3:**
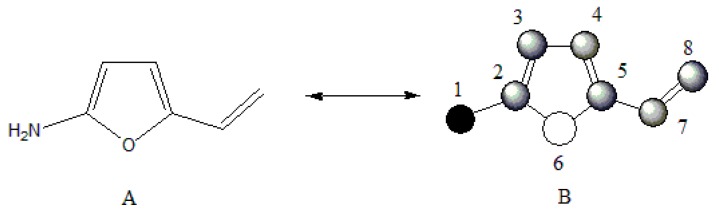
(**A**) Molecular structure of 2-amino-5-vinylfurane; (**B**) Corresponding graph with arbitrary numeration.

Let’s apply the previously discussed aspects in an example using the molecular structure of 2-amino-5-vinylfurane (Figure 3).

The event connected sub-graphs (S) considers the formation of the molecular structure (G) taking as base molecular fragments (sub-graphs with different orders and types according to Kier-Hall nomenclature, which is Path, Cluster, Path-Cluster and Chain) [10,11,16], By using the scheme of the Figure 2 and applying the event (S) for the order 1 (pairs of connected atoms or individual bonds), the following relations frequency matrix is obtained:

$$F=\left[\begin{array}{cccccccc} 1 & 1 & 0 & 0 & 0 & 0 & 0 & 0\\ 1 & 3 & 1 & 0 & 0 & 1 & 0 & 0\\ 0 & 1 & 2 & 1 & 0 & 0 & 0 & 0\\ 0 & 0 & 1 & 2 & 1 & 0 & 0 & 0\\ 0 & 0 & 0 & 1 & 3 & 1 & 1 & 0\\ 0 & 1 & 0 & 0 & 1 & 2 & 0 & 0\\ 0 & 0 & 0 & 0 & 1 & 0 & 2 & 1\\ 0 & 0 & 0 & 0 & 0 & 0 & 1 & 1 \end{array}\right]$$

For evaluating which bonds contribute in a greater measure, from a topological point of view, to the formation of the structure, the derivative over the pairs of atoms is calculated. For our previous example, the derivative values are:

$$\frac{\partial G}{\partial S}\left(1,2\right)=\frac{1-2\left(1\right)+3}{1}=2.00\;\frac{\partial G}{\partial S}\left(2,3\right)=\frac{3-2\left(1\right)+2}{1}=3.00$$

Analogically, for the rest of the connected pairs their derivative values are:

$$\frac{\partial G}{\partial S}\left(2,6\right)=3.00;\;\frac{\partial G}{\partial S}\left(3,4\right)=2.00;\;\frac{\partial G}{\partial S}\left(4,5\right)=3.00;$$

$$\frac{\partial G}{\partial S}\left(5,6\right)=3.00;\frac{\partial G}{\partial S}\left(5,7\right)=3.00;\;\frac{\partial G}{\partial S}\left(7,8\right)=1.00\;$$

Analyzing these derivative values, it is observed that the most influent pairs according to the chosen event (*i.e.*, the most contributing bonds in the formation of the molecular structure) are 2-3, 2-6, 4-5, 5-6 and 5-7, respectively. This is a logical result because the atoms 2 and 5 present the highest number of connections in the molecular graph G (Figure 3B). The GDI values obtained for all atom pairs can be organized as a matrix $D={\left[\frac{\partial G}{\partial S}\left(i,j\right)\right]}_{n\times n}$, where it is possible to obtain individual atomic local indices by adding all the values of the derivative for the atom *i*, which is equivalent to adding all the elements from each row or column from the D matrix. This atomic index $\Delta_{i}=\overset{n}{\underset{i=1}{\sum }}\frac{\partial G}{\partial S}\left(i,j\right)$ is a Local Vertexes Invariant (LOVI) [4,11,12,18,19]. The atomic indices Δ*_i_* for a given molecular structure with *n* atoms may be expressed as a LOVIs vector, (*V_L_*). In this sense, the LOVIs vector for the molecule of Figure 3 is: $V_{L}={\left[\begin{array}{ccc} \begin{array}{ccc} 2 & 8 & 5 \end{array} & 5 & \begin{array}{ccc} 9 & 6 & \begin{array}{cc} 4 & 1 \end{array} \end{array} \end{array}\right]}_{1\times 8}$.

However, *V*_L_ only takes into account information on the connectivity’s degree of the different atoms in the structure without considering the type of bonded atom. It is important to apply a weighting scheme to the atoms in a compound to yield a description much closer to the molecular structure reality. In the GDI this information is introduced through three different schemes, as it will be explained below.

#### 2.2.1. Atomic Differentiation

The matrix treatment of graphs does not guarantee the chemical differentiation of the elements in the G; thus, methods for differentiating the atoms of different nature in the molecule have been introduced. As a preliminary step, an atomic weight is assigned to each atom, through the expression:

(8) $$\vartheta_{i}=\frac{P_{i}}{\delta_{i}}$$

where, $\delta_{i}$ is the vertex degree for atom *i* in the molecular structure and *P_i_* is a property that characterizes each atom. With this definition a weight vector $V^{w}={\left[\vartheta_{i}\right]}_{1\times n}$ may be constructed, which contains information on each type of atom present in the molecule and positioned within a particular electronic environment. For practical purposes, the resulting values may be written as a row vector, column vector or as a W diagonal matrix, which is applied in three different schemes [10].

Weighting in the incidence matrix: A weighted incidence matrix may be obtained as a result of the multiplication of the Q incidence matrix with the weighted matrix W ($Q\;\times \;W=Q^{w}$). Subsequently, the graph’s derivation process is performed as previously described. A same result is obtained if we introduce the direct weighting of each atom (and/or atom-pairs) in the frequency matrix and ultimately yielding a weighted LOVIs vector (^w^V_L-f_). For the molecule in the Figure 3, the weighted LOVIs vector, using Pauling’s electronegativity as atoms weighting scheme, is VL−fw=[4.92, 11.29, 9.55, 10.61, 4.92, 3.60, 3.75, 0.83].

Weighting in the derivative matrix: Once we have the D matrix, it may be multiplied with $V^{w}\text{vector}$ and a new weighted LOVIs vector (^w^V_L-d_) is obtained. For molecular structure of 2-amino-5-vinylfurane, this vector would be VL−dw=[3.61, 14.40, 3.82, 10.57, 3.61, 1.27, 3.19, 0.85] .

Weighting in the LOVI vector: The product of the multiplication of V_L_ × W yields a new vector and its component also represent weighted LOVIs  (VLw=VL × W). For the molecule from Figure 3 VLw=[4.25, 5.10, 10.95, 5.74, 4.25, 6.38, 3.40, 1.27].

The definition of local atomic descriptors, following the previous prescription, is in harmony with one of the 13 properties proposed by Randic that new TIs should possess [4,6].

#### 2.2.2. Total and/or Local (Group or Atom-Type) Description

In analogy with the Molecular Orbitals Theory, which states that molecular orbitals can be obtained as a linear combination of atomic orbitals [20,21,22], MDs associated with the whole molecular structure (or to a group of atoms in the molecule) can be calculated through the mathematical invariants shown in Table 1.

In Table 1, the invariants (aggregation operators) are classified in four groups: (1) norms; (2) means; (3) statistical parameters and (4) “classic” invariants. Let’s compute some total and local invariants (unsaturated bonds and heteroatoms) for the 2-amino-5-vinylfurane molecule using the LOVIs of the unweighted atoms as an example:

Total Invariants: N_1_ = 40 (N_1_ means Manhattan norm (sum of atom-level descriptors (LOVIs), A (Harmonic Mean) = 5 and R (Range) = 8 (see Table 1 for more details).

Local Invariants over unsaturated bonds and heteroatoms: N_1_ (Unsaturated bonds) = 32 and N_2_ (heteroatoms) = 6.3245.

With this procedure, a great number of possibilities in the description of important chemical information on the molecular structure may be explored (see Table 1 for more details). In previous papers, all these invariants were introduced and applied to obtain total and local indices over groups and/or atom types, generalizing the traditional way for obtaining the global indices [10,11,12]. Other families of indices recently defined by our research group (Computer-Aided Molecular “Biosilico” Discovery and Bioinformatic Research (CAMD-BIR) International Network) also make use of these invariants and have been applied to many structure-activity/property relationship experiments with relevant results [3,17,23,24].

## 3. Structural and Physicochemical Interpretation of GDIs

The GDIs have been previously used in several theoretical applications, providing relevant results fundamentally in QSAR/QSPR studies. [10,11,12] However, little effort has been destined to the interpretation of these MDs in structural and/or physicochemical terms [10,11,12,16]. In this section, the results of different experiments designed to demonstrate the reliability of GDIs in structural and physicochemical hypotheses are presented. The GDIs interpretation will be developed using three different approaches, which will be detailed for every case study.

Regarding the computational details, the calculation of GDIs for all the databases was performed using the open-source, java-based software denominated as DIVATI (acronym for DIscrete DeriVAtive Type Indices), a new module of ToMoCoMD (Topological Molecular Computational Design) software [25].

QSAR/QSPR models were developed by using Multiple Linear Regression (MLR) with the MobyDigs software [26]. This program allows obtaining MLR equations using the genetic algorithm [27] as MD selection method. Additionally, it allows for several model validation procedures such as: internal cross validation (Q^2^_loo_), external validation (Q^2^_ext_), bootstrapping (Q^2^_boot_), and Y-randomization, as well as prediction analysis.

### 3.1. Structural Interpretation

Some of the 13 characteristics, proposed by M. Randic, that new TIs should ideally possess include: possible structural interpretation, isomers recognition, possibility for local definition, as well as present a correct dependence with size and gradual change with structural variations, among others [28]. These properties are closely related and all of them indicate the direct correspondence that should exist among topological indices calculated for a molecule and its structure. If this condition is achieved the MDs can, at least in principle, describe any chemical information extracted from the connectivity of the molecular structure.

All calculations for the structural interpretation experiments performed in this section were made for derivatives over pairs of atoms with respect to the connected sub-graphs event (each event yields different LOVI values, but the interrelations among them are similar), using the generalized matrix and Pauling’s electronegativity over the incidence matrix as the weighting scheme The LOVIs for each atom in the molecular structures employed in the present section, as well as the corresponding Minkowsky norm for *p* = 1(equivalent to the summation operator), the arithmetic mean, and the range (see Table 1) are depicted in Table 2, Table 3, Table 4 and Table 5.

#### 3.1.1. Differentiation among Homologs, Multiple Bond Codification and Positions

From Table 2, it can be inferred that LOVIs for all the different atoms decrease as one get closer to the center of the carbon chain, belonging to the considered linear alkanes and their homologs (for a sake of simplicity, the hydrogen atoms are no taken into account in the representation of the G).

The value of every atom of the chain also increases as one moves from an inferior homolog to a superior one, maintaining the regularity among the LOVIs of atoms in the same molecule. Besides, the total invariants shown in this Table reflect a regular increase in their respective values when the number of methylene groups –CH_2_– in the structure increases from one homolog to another. The increase of the electronic density and its position in a specific region can be also quantified using this mathematical description. It is observed that atoms connected by multiple bonds also increase their LOVI values when the number of bonds among them increases. Consequently, the total (global) invariants from this molecule increase when the number of atoms increases. As illustrated these total invariants can be used for differentiating position isomers because their values decrease in the measure that the multiple bond is more embedded in the molecular structure.

#### 3.1.2. Differentiation among Chain Isomers

In Table 3, are the results of an experiment analogous to the previous one but now using chain isomers of saturated hydrocarbons with six carbon atoms. Table 2 shows how the variations in the carbon chain from the molecule cause variations in the LOVIs values from each atom, according to the modifications experimented in the molecular structure, from one isomer to another.

It is interesting to note the peculiar variation of the different invariants when the quantities of ramifications increase, and consequently the length of the main chain decreases. Note that the invariants decrease their values, which is a logical result since LOVIs also experiment a decrease in their values in the measure that molecules are more ramified.

#### 3.1.3. Codification of the Presence of Different Functions

Until this point, only molecules comprised exclusively of carbon and hydrogen have been described. Here, the capability of GDIs for describing structures containing different organic functions is evaluated.

The organic functions play a fundamental role in the properties of bio-active molecules; therefore their description cannot be ignored. In Table 4 the results of the calculation of the atomic and total indices for a series of propane derivatives is summarized. As in the previous cases, the electronegativity according to Pauling’s scale is employed as label for differentiating the atoms in the molecule.

It is observed that when the electronegativity of atoms located in “X” position increases, the LOVIs values also increase and the total invariants increase in almost a regular way. It is interesting to detail that the increase observed in LOVI values, from a molecule to another, is proportional to the increase in the property used for characterizing the atoms. Besides, it should be pointed out that this increase in the LOVI values due to the presence of different X groups is identical to the summation of the increase of the rest of the chain.

#### 3.1.4. Cycle, Conjugation and Aromaticity

Table 5 shows a set of cyclic structures and some few acyclic chains. The purpose of choosing these systems is to determine if GDIs are able to retrieve information about the conjugation, aromaticity and the presence of cycles, during the numerical description of the molecular structure.

From a detailed analysis of data in Table 5, it can be observed that LOVIs from a cyclic structure are smaller in comparison to the values obtained for the corresponding acyclic structures with an isolated unsaturated bond. In the measure that the number of unsaturated bonds increase, the LOVIs values decrease, but these decrements are more significant when electronic conjugation is present in the structure, meaning that the aromatic molecules are characterized by the lowest values. The presence of heteroatoms in the systems increases the LOVIs values for carbon atoms compared to counterparts without heteroatoms. Moreover, atoms bound to heteroatoms present a smaller index value than the other ones, which is an indication that these atoms have a lower electronic density provoked by the direct interaction with a more electronegative atom. This result is consistent with the chemical reality of heteroatomic systems.

#### 3.1.5. Molecular Symmetry Described in Terms of GDIs

A more complete structural description is obtained when the symmetry of the molecules is incorporated in the description achieved for the GDIs as previously shown.

In this sense, Shannon’s Entropy is used as a symmetry index (see Equation (9)) [4]:

(9) $$SI=\frac{-\sum_{i=1}^{G}\frac{\rho \left(g_{i}\right)}{N}log_{2}\frac{\rho \left(g_{i}\right)}{N}}{log_{2}N}$$

where $\rho \left(g_{i}\right)$ is the cardinality of the equivalent classes, which in our particular case is the number of equivalent LOVIs and N is the total number of atoms in the molecule. The symmetry index may be interpreted as a measure of the bidimensional symmetry of an organic molecule, and it takes values between 0 and 1 (0≤SI≤1). A value of 0 corresponds to molecules with high 2D symmetry, whereas a value of 1 is associated with asymmetric molecules.

Table 6 shows the SI values computed for ten organic molecules, taking as base the cardinality of the LOVIs obtained with GDIs for derivatives over pairs of atoms with respect to the connected sub-graph event and by weighting the incidence matrix with Pauling’s electronegativity.

From the analysis of Table 6, it can be inferred that the resulting SI are logical and acceptable if it is considered that this index contains information on the symmetry of the molecules based on the local vertex invariants calculated for these 2D structures.

### 3.2. Reactivity Based on Geometrical Effects

Consider a molecule fragmented with respect to the connected sub-graph (S) for order 1. Note that the individual frequencies are the number of total connections for each atom in the model generated by the considered event. For this particular case (S event for order 1), the individual frequencies correspond to the number of sigma bonds for each atom. In electronic terms this frequency may be written as:

(10) $$f_{i}^{1}=V_{i}^{t}-H_{i}-\pi_{i}-n_{i}$$

(11) $$f_{i}^{1}=V_{i}^{HS}-\pi_{i}-n_{i}=\sigma_{i}$$

where, $V_{i}^{t}$, $H_{i}$, $\;V_{i}^{HS}$, $\pi_{i},\;n_{i}$, and $\sigma_{i}$ are the total valence, the number of hydrogen atoms bond, the valence number with suppressed hydrogen atoms, the quantity of π bonds, the number of lone pairs associated to atom *i* and the number of sigma bonds from the atom *i*, respectively.

According to the latter expression, it can be observed that the individual frequency of order 1 can be simply defined as the number of sigma bonds of atom *i*. In the case where the generalized frequency matrix (including *k*^th^-order sub-graphs, *k* > 1) is employed, the interpretation of the individual frequency holds but should be identified as the number of connections between atoms separated by distance *k*, depending on the model generated by the chosen event and the maximum fragment order.

In this sense, the derivative evaluated for frequencies of order 1, over a pair of bonding atoms may be written as:

(12) $$\frac{dG}{dS}\left(i\;j\right)=\left(V_{i}^{HS}+V_{j}^{HS}-2\right)-2\pi -n_{i}-n_{j}$$

(13) $$\frac{dG}{dS}\left(i\;j\right)=A-2\pi -n_{i}-n_{j}$$

For carbon atoms in an organic molecule Equation (13) can be simplified to:

(14) $$\frac{dG}{dS}\left(i\;j\right)=A-2\pi$$

By analyzing Equations (12)–(14), it is evident that the derivative codifies information on the total number of sigma type connections associated to a pair of atoms (*i j*) connected at a specific distance. In this sense, the derivative can be defined as measure of the “accessibility” of a given region of a molecule.

The LOVI for each atom *i* can be defined as the multiplicity of its sigma bonds affected or influenced by the respective surrounding multiplicities. This definition can be mathematically written as:

(15) $$\Delta_{i}=n\sigma_{i}+\overset{n}{\underset{j=1}{\sum }}\sigma_{j}-2n$$

The term 2n appears from the expression of the derivative and it has the effect of cancelling out the elements repeated in both groups of bonds.

Based on these concepts, the LOVI value of an atom can be regarded as the degree of participation of this atom in the formation of the molecular structure; therefore, the accessibility $\varepsilon_{i}$ to an atom can be evaluated as the inverse of its LOVI because it is proportional to the total area in which an atom is likely to be attacked by any external entity. In this sense, the interaction capacity of two atoms located in different molecules can be evaluated as the product of their accessibilities $\left(\rho_{ij}=\varepsilon_{i}\varepsilon_{j}\right)$, defining the interaction capacity of an atom *i* with any other atom *j* as the addition of accessibility contributions of the atom *i* with the rest $\left(I_{i}=\overset{n}{\underset{j=1}{\sum }}\rho_{ij}\right)$. Generalizing this idea, the interaction capacity of two molecules (Ω), understood as the total contribution of all the individual capacities of the constituent atoms determined from their accessibilities, should be proportional to the interaction probability of two molecules, which can be mathematically expressed as:

(16) $$P\sim \Omega =\hat{M}\left(\overset{n}{\underset{j=1}{\sum }}\rho_{ij}\right)$$

where, $\hat{M}$ is an operator, which involves the operation of the individual interaction capacity from each atom (*M* would be the sum, product, *etc.*), *P* is the real probability of interaction between two molecules and Ω is the theoretical probability evaluated by taking as base the LOVIs from each atom. The real value of the intermolecular interaction probability *P* depends on the structures (symmetry, form, size and distribution). These structural parameters are carefully quantified by GDIs as was showed in the previous epigraph.

In Collisions Kinetic Theory, the magnitude known as the Steric Factor (F), is considered as a consequence of the analysis of incongruences between theory and the experimentation results and it expresses the probability of the corresponding geometrical configuration during interactions [29]. Posterior deductions showed that the Steric Factor from Collisions Kinetic Theory is analogous to the $e^{\Delta S^{\neq }/R}$ term, which is related with the probability of existence of a viable configuration able to interact with another specie [29] and $\Delta S^{\neq }$ is the variation of entropy of the active complex and R a constant $\left(R=8.314\text{ J}\cdot {\text{mol}}^{-1}\cdot K^{-1}\right)$.

The LOVIs, their inverses, and the parameters introduced in both kinetic theories, have a direct relationship with the probability of intermolecular interaction based on structural configurations during the interaction. In this sense, it is possible find analogies between these kinetic parameters and the local and total indices derived from the corresponding mathematical procedures implicit in GDIs calculation.

To evaluate the veracity of the aforementioned ideas, second order dimerization reactions, in gaseous states were studied as an example. Table 7 summarizes the ΔS^#^ data for each studied reaction [30].

In each case, the total interaction capacity Ω was evaluated and it was related with the activation entropy for the process of dimerization of diverse unsaturated molecules, yielding a correlation of 97.58%. The operator taken to evaluate the totality of the atomic contributions was the standard deviation, which is a measure of the dispersion of the values.

The activation entropy is a parameter associated with the probability of an adequate interaction between two spaces, taking as reference the structural organization of the created transition state, which is product of a suitable interaction. In this sense, it is logical to find a strong relation between parameters obtained from GDIs calculation and this magnitude. This example can be taken as base for understanding the molecular graph derivative from a kinetic point of view, which is closely related with the possibility of interaction of the molecules based on their corresponding geometrical structures.

#### 3.2.1. Accessibility as Measure of the Interaction’s Capacity

Accessibility is defined as the possibility of entrance or access capacity, in this case, to a specific region in a molecule. The Kier and Hall [31,32] molecular connectivity indices have been recently studied by Estrada in terms of the Relative Bond Accessibility Area (RBA), *C_ij_*, which is expressed in square Randics (*R*^2^) [33,34]. The reactivity of an atom is related with its accessibility, and the accessibility can by quantified from topological features of the molecular structure that contains this atom [35,36].

As was mentioned in the previous section, the inverse of a LOVI may be considered as a measure of the availability of a particular zone of a molecule (fully characterized by a pair of atoms) to interact with an external agent. From this definition, it can be stated that this MD is closely related to the steric effects controlling molecular reactivity or simply steric reactivity. The steric reactivity associated to a pair of bound atoms can be evaluated as $\left(\frac{1}{\Delta_{i}\Delta \Delta_{j}}\right)$, where $\Delta_{i}$ is the LOVI of atom *i*. In Table 8, steric reactivity indices computed for a collection of molecules are summarized together with RBA in *R*^2^ calculated by Estrada for these molecules [33,34].

**Figure 4:**
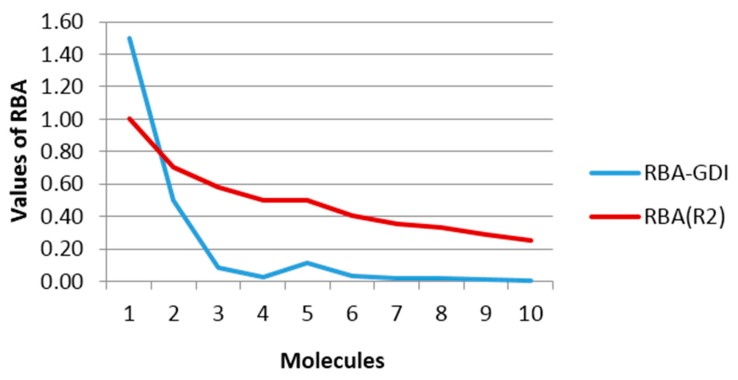
Graphical behavior of the steric reactivity based in LOVIs calculated by GDIs and the Relative Bond Accessibility Area (RBA) proposed by Estrada for evaluating the accessibility.

Both sets of data from Table 8 are plotted in Figure 4, where a qualitative agreement between the two curves may be observed. Note that for the pair of atoms (a,b) in the molecules **4** and **5** (2,2-dimethylpropane and butane, respectively) the same RBA values were obtained using the connectivity index, while the evaluation of the steric reactivity with GDIs calculations yielded better differentiation more consistent with the chemical reality because it is anticipated that the a–b bond in the 2,2-dimetylpropane molecule would be less accessible due to greater steric hindrance than the a–b bond in the butane molecule. This challenge of index degeneration in situations like these is not encountered with GDIs.

Another important aspect is the infinite value obtained for the trivial case of the ethane molecule. Although this value is useless for further statistic or algebraic developments, it is logical if we take into account that it is evaluating the accessibility of an entity external to a particular bond in each molecule. With this idea it plausible that the ethane molecule has infinite accessibility possibilities given that it is constituted by only one bond (G with suppressed H-atoms were considered in all cases). To visualize this effect in Figure 4, an R-GDI value equal to 1.5 was arbitrarily assigned to this molecule to give the idea that it is superior and thus allowing for the visualization of the regularities of the rest without affecting the scale. Note, however, that this does not mean that GDIs would not have the capacity of codifying the structure of one molecule as simple as ethane, because this infinite value is only given for the order 1, which is the configuration used for simplifying the physical interpretation of the GDIs that may later be generalized to more complex systems (*i.e.*, using sub-graphs of superior orders in the event S, derivatives over *n*-elements, using other events, and so on).

#### 3.2.2. Specific Reaction Rate and Its Relation with GDI

The specific reaction rate constant is the speed of chemical transformation when the concentration of all the reagents is equal to 1 mol/L [29,30]. Thus, the rate constant is a magnitude proportional to the reactivity of the molecules that participate in a reaction, with the temperature of the system being constant [29].

In the previous sections the derivative over a pair of bonded atoms was defined as the quantity of sigma connections of both atoms and was expressed as a difference of electronic contributions that express the part of the total electronic density destined to connections or sigma bonds. In this sense, the derivative over a pair of atoms *i and j* can also be understood as a measure of the interaction capacity of a bond with a neighboring molecule.

**Figure 5:**
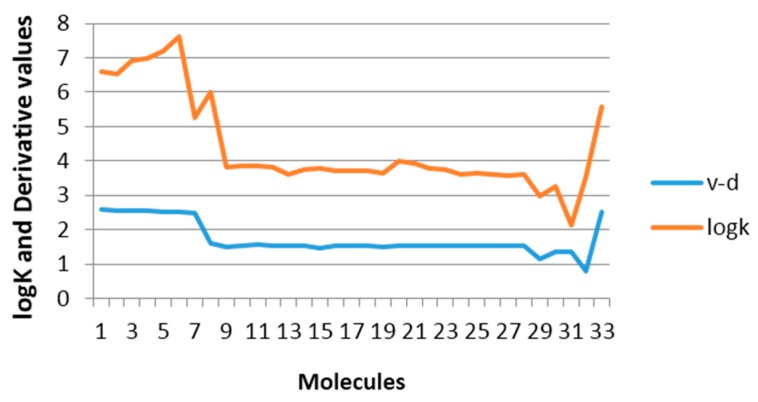
Regularity in the Derivative (over exocyclic double bond) variation and the log*K* values.

To evaluate the previous affirmation a data comprised of 34 derivatives of 2-vinylfuranes was employed, for which the specific rate constant for the nucleophylic addition to the double exocyclic bond from this molecules with mercaptoacetic acid has been reported [2]. The best one variable regression shows that there exists a moderate relation between the property and the GDIs. However, taking into account that the best models reported by other authors for describing this property use seven variables [2,37], it is noteworthy that with only one descriptor (derivative over the double exocyclic bond) it is possible to explain approximately 80% of the variance of the experimental property. Figure 5 shows the correspondence between the derivative values and the experimental values from log*K*.

### 3.3. Interpretation of GDIs in Electronics Terms

The nature of the atoms and the molecules is determined by their electronic structure [21], thus, by describing the dynamics, distribution and the electronic energy of these systems, it is feasible to establish a useful nexus for the better understanding of molecular structures and/or the methods used to codify them.

**Figure 6:**
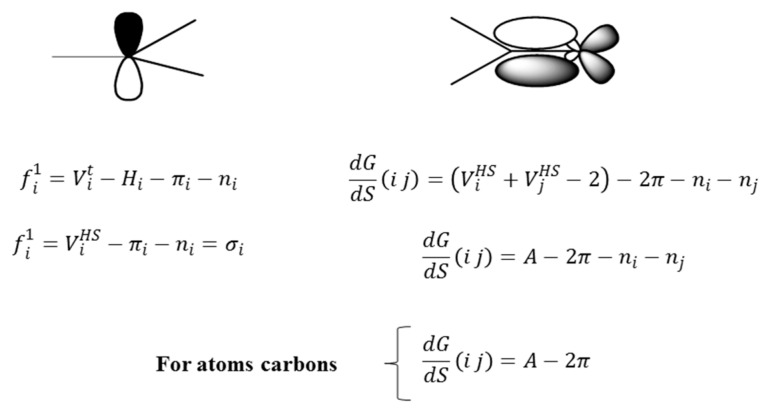
Own Frequency and duplex derivative in electronic terms.

The detailed structural analysis in the previous epigraph showed that there is a relationship between GDIs and the influence of the electronic richness of atoms bound to a specific center in a molecule. Moreover, the obtained result demonstrated the possibility of a physicochemical interpretation in kinetic terms, on the basis of the quantification of the interaction potential of a molecule described by means of GDI and LOVIs obtained in geometrical framework. All these aspects are easily assimilable if we take into account the individual frequencies and the derivatives over atom pairs expressed in electronic terms as explained in the previous section. Figure 6 illustrates the electronic decomposition of the individual frequencies of order 1 and the derivatives of order 1 for a pair of bonded atoms.

Up to this point, only the quantity and distribution of bonds in the molecular structure has been taken into account for the GDIs interpretation. However, the knowledge about these bonds and their distribution around each vertex determines the electronic density in the environment of each atomic nucleus in the molecular structure, and thus motivating a deeper analysis of the GDIs from a Quantum Mechanics perspective.

From Figure 6, the orbital description of the frequencies allows for two observations: (1) if the frequency is equal to the number of sigma bonds of an atom, it then also quantifies the hybridization of the atom and the symmetry of the sigma electronic distribution and (2) the parameters to the left suggest a separation of the electronic terms corresponding to the quantification of σ, π and non-shared electrons, which allows for the evaluation of the separated influence of these types of electrons, as it will be corroborated in the next part of the present study regarding the relation of the frequencies with Hückel’s free valence. It can be pointed out that only the frequency of order 1 is similar to the number of sigma bonds belonging to an atom. Nonetheless, it is anticipated that a similar concept holds in an abstract model based higher order frequencies obtained according to different fragmentation approaches.

For the case of the derivative, it can by described as a quantity related to the part of the total valence of the bonding atoms, which is destined to the establishment of connections in a molecular network. Equally, the separation of the π electrons offers a differentiated treatment to each type of electrons (it makes reference to the electrons that form part of the covalent bonds, π- or σ-type and non-shared electrons), considering the basic differences among their characteristics and expressing one in function of others, which shows the relation among them.

The fact that both the frequency and the derivative can be can be expressed in terms of the distribution of electrons around one atom or bond approximates to the idea of chemical reactivity, now understood from the perspective of the electronic interaction capacity. In next sections, carefully designed experiments will show the strength of these basic local descriptors from the discrete derivative algorithm in describing the electronic characteristics of atoms and molecules.

#### 3.3.1. GDIs, Chemical Reactivity and Relation with Hückel’s Free Valence

A possible approximation for studying the chemical reactivity is to determine the degree in which the atoms in a molecule are united to the adjacent atoms, which is relative to their theoretical bonding capacity [38], The degree in which one atom is united to its neighbors can be calculated summing all the values of the bond orders of that atom. If the sum of all the bond orders is subtracted from the value of the highest bonding capacity, we would obtain the free valence:

(17) $$F_{r}=\text{Highest bonding capacity}-\overset{n}{\underset{j=1}{\sum }}\rho_{ij}$$

For conjugated hydrocarbons, the free valence index can be evaluated as:

(18) $$F_{r}=4.732-\overset{n}{\underset{j=1}{\sum }}\rho_{ij}$$

where, $\rho_{ij}$ is the bond order for the atoms (*i j*), determined by Hückel molecular orbitals method [38,39].

The individual frequency of each atom evaluates the number of connections of an atom in a specific model generated by an adequate event for describing the molecular structure. In the particular case of the event S, if only the order 1 matrix is taken into account, it is possible to relate this frequency with the quantity of sigma bonds of an atom.

Reorganizing the terms for the individual frequency shown in the Figure 6, we obtain:

(19) $$V_{i}^{T}-f_{i}^{1}=H_{i}+\pi_{i}$$

The term to the left of the Equation (19) describes the part of the total valence dedicated to forming π type bonds. The right term shows the number of π reactive electrons and the number of H-atoms bonded to each carbon. Both members from that equation show huge similarities with the Equations (17) and (18), although from different optics but explaining the same characteristic: chemical reactivity of each carbon atom in the molecular system, therefore, it should be hoped that:

(20) $$F_{r}\sim \left(4-f_{i}\right)=\left(H_{i}+\pi_{i}\right)$$

Taking into account the equation 19, a study was performed on the behavior of the free valence calculated by the Hückel molecular orbitals method for specific atoms with different environments in a dataset composed of 19 conjugated hydrocarbons, with some of them being aromatic. This chemical dataset was primarily used by Kier and Hall [39] with the goal of finding a relationship between the free valence and the Electropologic State index, which is a descriptor regularly employed in correlations with electronic properties of different molecular systems and known to yield good results [39,40].

**Figure 7:**
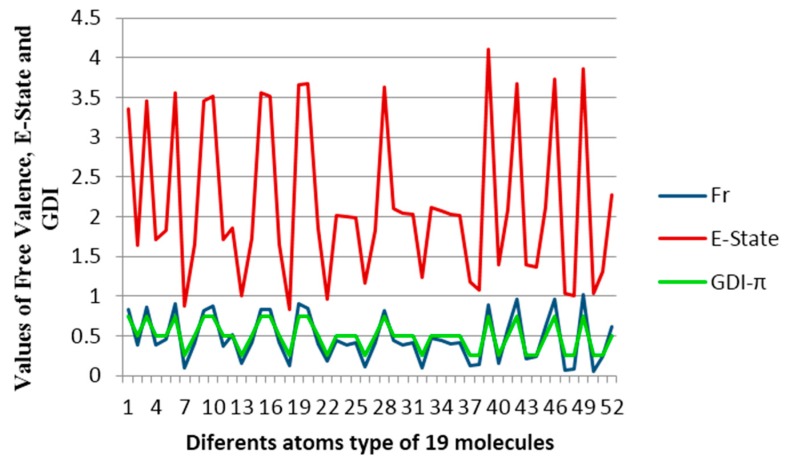
Behavior of the free valence, the E-States and GDIs for atoms from 19 conjugated molecules.

Figure 7 shows the free valence calculated by the Hückel’s molecular orbitals method (blue line), the Electropologic State index (red line) and the derivative indices taking as base the individual frequencies of the atoms. A uniform variation between the values obtained for Hückel’s free valence and the GDIs is observed and this evidently shows that the GDIs quantify electronic environments of atoms in the molecules and reinforcing the understanding of the GDIs in reactivity terms. In previous studies, Kier and Hall grouped the atom types in these molecules in three categories: carbon atoms with one hydrogen, carbon atoms with two hydrogens and carbon atoms without hydrogen [39]. Likewise, a linear relationship is found in the present experiment:

(21) $$F_{r}≅1-f_{i}^{1}+C^{t}$$

where, $C^{t}$ is the proportionality constant which adopts different values for each type of atom from the system and it allows finding reactivity values closer to the values evaluated by Huckel’s method. Table 9 shows the six types of atoms that can appear in conjugated systems conformed by only one carbon and hydrogen.

The Equation (21) shows a simple relation to evaluate Hückel’s free valence taking as base the order 1 frequencies with the S event. It is obvious that in the Expression (21), the goal is not the substitution of the real calculation of Hückel’s free valence, but the use of this expression allows establishing in a faster and simple way an estimate of the chemical reactivity of any carbon atom in a conjugated molecule without evaluating the bond order, for which it is necessary to know the coefficients of each atomic wave function that participates in the linear combination to form the molecular wave function.

Another advantage of the Equations (20) and (21) is that they demonstrate a clear existence of a nexus between the electronic environment of the atoms and their corresponding atomic indices evaluated with derivative indices, reaffirming the notion that graph derivative indices describe electronic properties of atoms in a molecule and consequently their electro-chemical reactivity.

#### 3.3.2. Electronic Interpretation

In the beginnings of 1950s, it was discovered that the resonance frequency of a nuclide depends not only of its magnetogyric ratio and the intensity of the magnetic field B_0_, but that it also depends of the electronic environment where the nuclide is located [41]. For one nuclide in a specific substance, there will be as many resonance frequencies as the electronic environments. This phenomenon known as chemical displacement (shift), is the base of the NMR (Nuclear Magnetic Resonance) [22,41] chemical applications. The chemical shift is a descriptor of the electronic characteristics of each atom from a molecule. In the following experiments we intend to find linear relationships between the chemical shift of some active nuclides in NMR, with the goal of discovering in what measure the electronic information of atoms and molecules is codified in the structural descriptions based on the GDI concept.

##### QSPRs of Chemical Shift of ^17^O-NMR for Aldehydes and Ketones

For this analysis a data of aldehydes and ketones was used, which have been previously studied by Kier and Hall [40] with the Electropologic State index. All molecules are aliphatic and their chemical shifts of the ^17^O have been reported in the literature (see Table 10).

**Figure 8:**
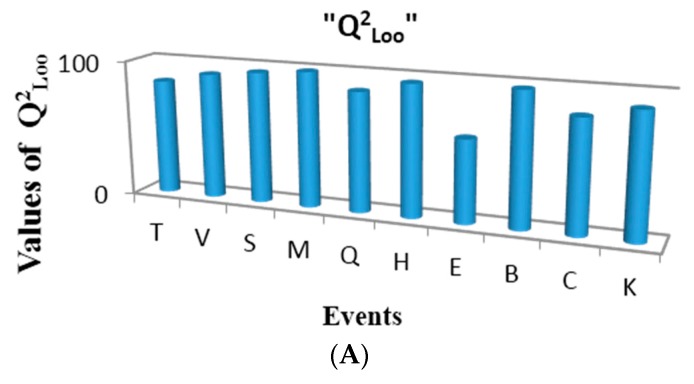

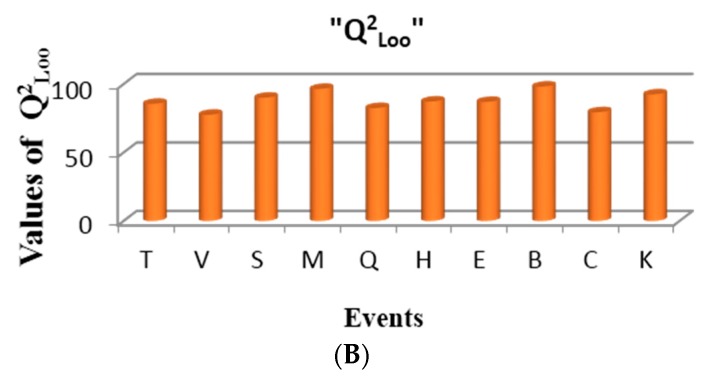
Development of the one-variable linear regression models obtained for each event: (**A**) Ethers; (**B**) Aldehydes and Ketones.

The variables used for linear regression were obtained by calculating the duplex derivative with respect to 10 different events (using chemical, physical and graph-theoretical atom-labels) and several norms, means, statistical and classic invariants as total and local MDs. For this experiment the MobyDigs software was used [26]. Figure 8A shows the performance of one variable regressions built for each of the events in the present study, according to the cross validation parameter Q^2^_Loo_. As can be observed, the events with the best correlations for the studied property are Sub-Structure (B) and Multiplicity (M), respectively. This observation is logical considering the fact that first one is a fingerprint-based event [3,16], and it conforms the incidence matrix only with substructures with functional groups and/or atom types of chemical interest, while the second one is a topological description of the connections at one step topological distances and their multiplicities (simple, double and triple bonds between pairs of atoms in a molecule) [3,16]. These two events yield matrices that reflect the electronic richness of the molecule, fragmented in individual bonds and their multiplicities. The best regression (based on the sub-structure event) obtained in the present study is shown below (see Equation (22)):

(22) δ=592.94(±1.58)−8.62(±0.38)[AN/In(IS)]BDf

*R*^2^ = 98.65%, Q^2^_Loo_ = 98.1%, Q^2^_Boot_ = 98.21%, s = 2.304, s_cv_ = 2.41, y_sc_ = −0.051, F = 512.25.

As it can be observed from the statistics of this equation, a good correlation (*R*^2^ = 98.65% and Q^2^_Loo_ = 98.10%) is obtained between the calculated MDs and the experimental chemical shift for ^17^O. An analysis of the descriptor contained in this model shows that it expresses arithmetic mean of the LOVIs of the carbon and oxygen from carbonyl group (IS: unsaturated bond), weighted by the valence degree, which is a topological expression of each atom [4]. This is a logical result taking into consideration that the value of the chemical shift of oxygen mainly depends on the electronic environment of this atom as well as of the influence of the electronic density of its unique adjacent atom (carbonyl carbon); therefore, the average is a direct quantitative measure of the electronic richness in the model, which is the main factor that influences the chemical shift of the nuclide of ^17^O.

##### QSPRs of Chemical Shift of ^17^O-NMR for Eithers

A similar study was performed using a dataset composed of 10 aliphatic ethers (Table 11), for which the chemical shift of ^17^O-NMR was reported [40]. The best one-variable model obtained in this study is shown below (Equation (23)):

(23) δ=105.54(±2.82)−3.64(±0.109)[AT/In(HT)]MDf

*R*^2^ = 99.28%, Q^2^_Loo_ = 98.94%, Q^2^_Boot_ = 99.05%, s = 3.588, s_cv_ = 3.891, y_sc_ = −0.046, F = 1102.54.

As can be seen, this model is statistically robust (*R*^2^ = 99.28% and *Q*^2^ = 98.94%). It is especially interesting that the variable that best correlates with the modeled property is the one that uses the topological polar surface area (T) to weight the oxygen LOVI. This property is an expression of the electronic and steric environment of the oxygen nucleus and the LOVI explains the influence of the groups adjacent to oxygen. Therefore, it is logical that this local atomic index has greater influence in modeling the studied property.

Figure 8B shows the performance of one variable models built for the events in the present study, according to the cross validation parameter Q^2^_Loo_. As can be observed, the best events for describing the electronic properties of atoms in these molecules are: multiplicity (M) and sub-structure events (B), respectively. Good performance is also achieved with connected sub-graphs (S) and Sach’s sub-graphs (H).

Table 11 shows the values of the variable that best correlates with the mentioned property. In the measure that chemical shift values increase, the LOVIs values decrease in a regular way, having a negative contribution in the model.

This linear relationship between the LOVIs from the oxygen atom and its chemical shift in NMR implicates that the LOVIs from the oxygen atom codify electronic information varying in an almost uniform way with the chemical shift values.

##### Comparison between Real Spectrums and Atomic GDIs for Alkanes

**Figure 9:**
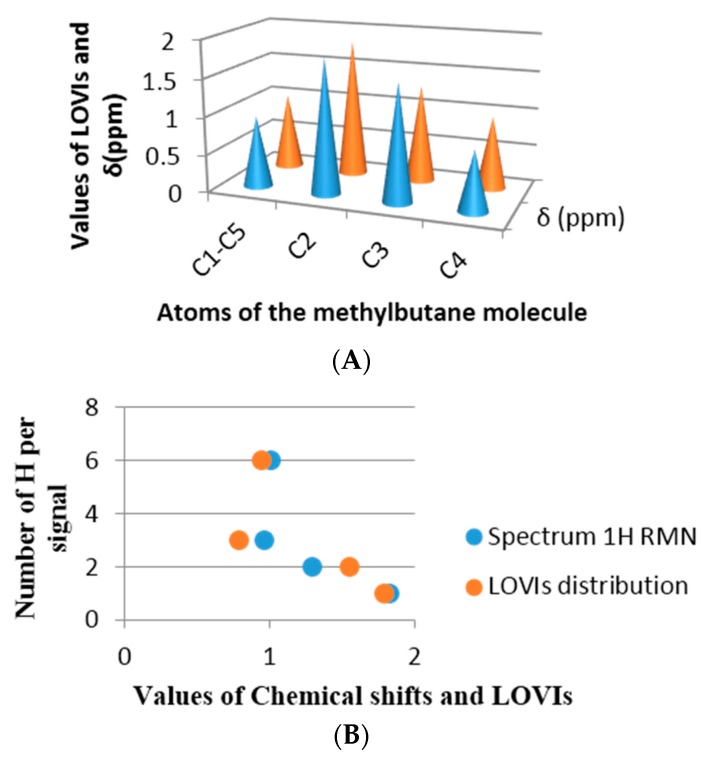
Methylbutane. (**A**) Equivalence between LOVIs values and the chemical shift in ppm; (**B**) Quantity of protons that provoke the signal *vs*. LOVIs and ppm.

To comprehend better the relationship of the GDIs with the electronic properties of the atoms of a molecule, some simple alkane molecules have been selected and their protonic Nuclear Magnetic Resonance spectrum (^1^H-NMR) predicted. Posteriorly, these are superimposed with the LOVIs values computed for the atoms in each molecule. The spectra were predicted using the ChemDraw program [42] and in both examples a good estimation was achieved. The selected molecules were methylbutane and 2,2,3-trimethylpentane, both molecules have all their sp3 carbon with different environments. Figure 9 shows the correspondence obtained between the chemical shift in ppm from each proton and the inverse of the LOVI value for each carbon corresponding to that proton (or group of protons). It can be observed that there is an unequivocal numerical proximity between both groups of values.

**Figure 10:**
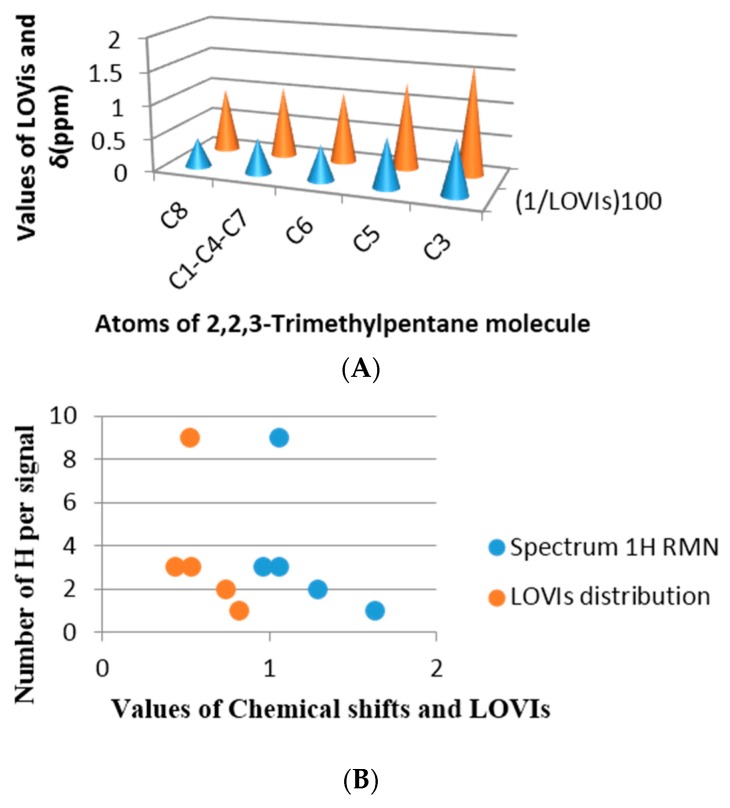
2,2,3-Trimethylpentane. (**A**) Equivalence between LOVIs values and the chemical shift in ppm; (**B**) Number of protons that provoke the signal *vs.* LOVIs and ppm.

If the chemical shift is a numerical expression for electronic density of a nuclei as well as the surrounding electronic environment, influenced by the electronic richness of the adjacent atoms, then a linear relationship between the chemical shift and the GDIs may be found, which means that our MDs codify steric and electronic information of the atoms in the molecules. Figure 9B and Figure 10B in each previous case are an approximation of the real spectrum (without taking into account the signal’s multiplicities), where the similarity of both spectra may be observed: the spectrum obtained from the chemical shift and the one obtained from the calculated GDIs. However, when a more realistic spectrum is needed, additional considerations are required: Calculation of the integrated intensity and multiplicity.

The Integrated Intensity (quantities of hydrogen bonded to the atom (*i*), which provokes the signal) is computed according to the Equation (24):

N_H_ = 4 – δ*i* (24)

On the other hand, the signal’s Multiplicity is determined by Equation (25):

M*_i_* = Σ(4 − δ*j*) + 1 (25)

where δ is the vertex degree of an atom and the atoms in the sum are those that possess derivative values different from zero with the atom *i*, for the order 1, according to the Connected Sub-graph (CS) or Multiplicity (M) event.

##### Description of Global Electronic Properties. Energy of Resonance

The resonance energy or stabilization energy by resonance is the difference between the energy corresponding to the structure with double (or triple) rigid bonds established in positions located in molecules with alternate unsaturated bonds (the most probable Kekule’s structure) and the real energy of the substance. The latter (*i.e.*, real energy) is less than the former and this decrement is associated with electronic delocalization [43,44]. In this study the correlation between the resonance energy from a dataset composed of 17 aromatic molecules and values calculated by GDIs was determined using several atomic labels (chemical, physical and graph-theoretical atomic properties) and 10 events. The existence of a linear correlation between the previous mentioned property and GDIs implies that they are able to characterize electronic densities and their delocalization capacity (which would corroborate with the study in Epigraph 3.1.4).

The best one and two variable models together with their corresponding statistical parameters are shown in Equations (26) and (27), respectively:

(26) ER=−4.16(±2.77)+0.48(±0.02)[N1L/In]MDf

*R*^2^ = 98.25, *s* = 5.286, *Q*^2^_Loo_ = 97.41, *s*_CV_ = 6.049, *Q*^2^_Boot_ = 97.60, *y*_sc_ = −0.038, *F* = 844.03.

(27) ER=−4.24(±2.22)+8.69(±1.58)[CC/PdN1(K)]HDf−0.94(±0.02)[AG/PlC5(S)(IS)]HDf

*R*^2^ = 99.24, *s* = 3.613, *Q*^2^_Loo_ = 98.99, *s*_CV_ = 3.764, *Q*^2^_Boot_ = 99.01, *y*_sc_ = 0.017, *F* = 910.02.

The satisfactory statistical parameters from these models demonstrate the close linear relationship between GDIs and the resonance energy for this group of molecules. The Equation (26) with only one variable is able to explain more than the 98% of the variance of the property. Table 12 shows the experimental and calculated values with the Equations (26) and (27), and their corresponding residuals. It is interesting to point out that the invariant that best correlates with this electronic property is the norm 1, which is the linear combination of the individual LOVI values.

In the previous experiment it was demonstrated that the atomic index values have a close relationship with the electronic properties of atoms in their molecular environment; therefore, as expected one total descriptor such as the norm 1 (N_1_, see Table 1 for more details) adequately codifies all the contributions and it expresses the electronic characteristics of the molecule, showing also a good correlation with the resonance energy, which is a property that express the electronic behavior product of conjugation. The two variable model contains a local descriptor for atoms forming part of unsaturated bonds. This variable quantifies the electronic effects of all the atoms with these characteristics in the molecule. Both descriptors from Equation (27) were derived with respect to the Sach’s sub-graphs (H) event, because its fragments only take into account the sub-graphs of order 1 and ring sub-graphs [3,16,45,46,47].

**Figure 11:**
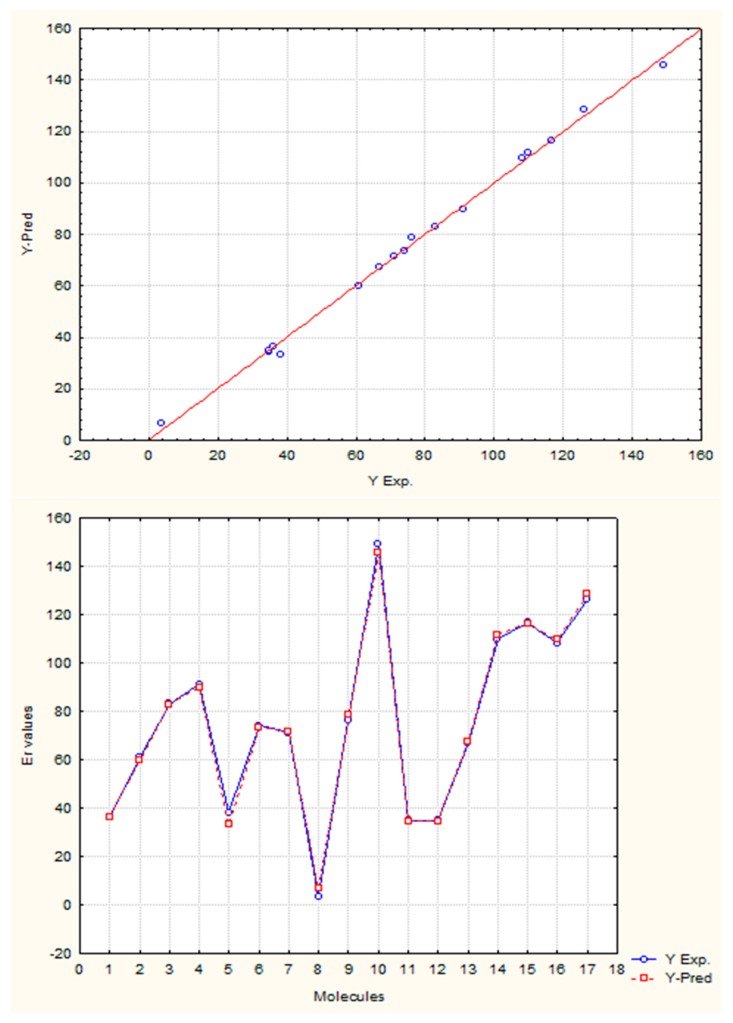
Regression and prediction graphs for the Equation (27).

The present study adds value to the previous experiments, which show the capacity of quantifying the electronic environment in atoms and molecules with this mathematical approach. The Figure 11 shows the regression graphs and the comparative behavior between experimental and theoretical values from the Equation (27).

## 4. Conclusions

The capacity of the GDIs to extract relevant structural information of organic molecules and consequently express it in terms of atomic local and total values has been demonstrated. It was observed that GDIs codify information on molecular symmetry, allow for the characterization of molecular structures with different sizes, are sensitive to structural ramifications, adequately characterize differences in the electronic densities of atoms at different positions in the structures, including cases of conjugated systems. Additionally, these TIs take into account the presence of heteroatoms and how they affect the electronic environment of the molecule.

Taking in consideration the regularity and coherence found among GDIs and each one of the structures described by this method, it may be affirmed that the GDIs possess direct structural interpretation allowing for greater comprehension of the chemical information codified [28]. Additionally, it was demonstrated that there exists a relationship between GDIs and the geometric reactivity, seen as a combination of the accessibility to the molecular structure and the activation entropy in the interaction process.

The transformation of the frequency and derivative parameters in electronic terms revealed that GDIs locally codify the characteristics of the electronic distribution of atoms and bonds and can be expressed as the electronic reactivity of atoms and molecules. The application of several mathematic operators to obtain global and local indices over a group of atoms, as well as a combination of these in linear models are an expression of more complex molecular properties such as the energy, analogous to the function of the operators employed in quantum mechanics.

## Figures and Tables

**Table 1:** Invariants functions employed to derive molecular descriptors (total and local) from local vertex invariants (LOVIs).

No.	Group	Name	ID	Formula ^a^
1	**Norms** (Metrics)	Minkowski norms ( *p* = 1) Manhattan norm	N1	$N_{1}=\sum_{i=1}^{n}\left|L_{i}\right|$
2		Minkowski norms ( *p* = 2) Euclidean norm	N2	$N_{2}=\sqrt{\sum_{i=1}^{n}{\left|L_{i}\right|}^{2}}$
3		Minkowski norms ( *p* = 3)	N3	$N_{3}=\sqrt[{3}]{\sum_{i=1}^{n}{\left|L_{i}\right|}^{3}}$
4		Chebyshev distance	NI	NI=limn→∞[∑i=1nLin]1n
5	**Mean** (first statistical moment)	Geometric Mean	G	$G=\sqrt[{n}]{\prod_{i=1}^{n}L_{i}}$
6		Arithmetic Mean (Power mean of degree α = 1)	M (or M_1_)	$M_{\alpha }={\left(\frac{L_{1}^{\alpha }+L_{2}^{\alpha }+\ldots +L_{n}^{\alpha }}{n}\right)}^{\frac{1}{\alpha }}$
7		Quadratic Mean (Power mean of degree α = 2)	P2 (or M_2_)	
8		Power mean of degree α = 3	P3 (or M_3_)	
9		Harmonic Mean (Power mean of degree α = −1)	A (or M_−1_)	
10	**Statistical** (highest statistical moments)	Variance	V	$V=\frac{\sum_{i=1}^{n}{\left(L_{i}-M\right)}^{2}}{n-1},\;$ *M*: arithmetic mean
11		Skewness	S	$S=n\left(X_{3}\right)/\left[\left(n-1\right)\left(n-2\right){\left(DE\right)}^{3}\right]$, *n*, number of vertices. $X_{3}=\sum_{i=1}^{n}{\left(L_{i}-M\right)}^{3}$, *M*: arithmetic mean, DE, standard deviation
12		Kurtosis	K	$K=\frac{\left[n(n+1)(X_{4})-3(X_{2})(X_{2})(n-1)\right]}{[(n-1)(n-2)(n-3){\left(\mathrm{DE}\right)}^{4}}$, *n*, number of vertices; $X_{j}=\sum_{i=1}^{n}{\left(L_{i}-M\right)}^{j}$, *M*: arithmetic mean, DE, standard deviation
13		Standard Deviation	DE	$DE=\sqrt{\frac{\sum_{i=1}^{n}{\left(L_{i}-M\right)}^{2}}{n-1}}$
14		Variation Coefficient	CV	CV=DEM
15		Range	R	$R=L_{max}-L_{min}$
16		Percentile 25	Q1	$Q_{1}=\left(\frac{N}{4}+\frac{1}{2}\right),$ *N*: *L_i_* number
17		Percentile 50	Q2	$Q_{2}=\left(\frac{N}{2}+\frac{1}{2}\right),$ *N*: *L_i_* number
18		Percentile 75	Q3	$Q_{3}=\left(\frac{3N}{4}+\frac{1}{2}\right),$ *N*: *L_i_* number
19		Inter-quartile Range	I50	$I_{50}=Q_{3}-Q_{1}$
20		Maximum value	MX	MX = *L_i_* max
21		Minimum value	MN	MN = *L_i_* min
22	**Classical** (Invariants)	Autocorrelation	ACk	$AC_{k}=\sum_{i=1}^{n}\sum_{j\geq 1}^{n}L_{i}\times L_{j}\cdot \left[\delta \left(d_{ij},k\right)\right]$, k=1,2,…7
23		Gravitational	GIk	GIk=1n∑i=1n∑j=1nLiLjdijk·[δ(dij,k)], k=1,2,…7
24		Total sum at lag k	TSk	$TS_{k}=\sum_{i=1}^{n}\sum_{j=1}^{n}L_{ij}\cdot \left[\delta \left(d_{ij},k\right)\right]$, k=1,2,…7
25		Kier-Hall connectivity	CNm	KHtm=∑i=1n(∏i=1nkLi,w)kλ where, *k* is the number of sub-graphs, *n_k_* is the number of atoms in a fragment, λ is equal to ½, *m* and *t* are the sub-graph order and type, respectively
26		Mean Information Content	MI	$MI=-\sum_{i=1}^{A}\frac{N_{g}}{N_{0}}log_{2}\frac{N_{g}}{N_{0}}$ where, *N*g is the number of atoms with the same LOVI value; *N*o, is the number of atoms in a molecule
27		Total Information Content	TI	$TI=N_{0}log_{2}N_{0}-\sum_{g=1}^{G}N_{g}log_{2}N_{g}$
28		Standardized Information Content	SI	$SI=\frac{TI}{N_{0}log_{2}N_{0}}$
29		Electrotopological state (E-state index)	ES	$SI=I_{i}+\Delta I_{i}=I_{i}+\sum_{j=1}^{n}\frac{I_{i}-I_{j}}{{\left(d_{ij}+1\right)}^{2}}\;$ where, *I_i_* is the intrinsic state of the ith atom and Δ*I_i_* is the field effect on the ith atom calculated as perturbation of the *I_i_* of ith atom by all other atoms in the molecule, *d_ij_* is the topological distance between the ith and the jth atoms, and *n* is the number of atoms. The exponent *k* is 2.
30		Ivanciuc-Balaban Type-Indices	IB	$J_{k}=\frac{n^{2}\Delta B}{n+C+1}\sum_{i=1}^{n-1}\sum_{j=i+1}^{n}\alpha_{ij}{\left(L_{i}\times L_{j}\right)}^{-\frac{1}{2}}$ where, the summation goes over all pairs of atoms but only pairs of adjacent atoms are accounted for by means of the elements α*_ij_* of the adjacency matrix. The *n*, *B*, and *C* are the number of atoms, bonds, and rings (cyclomatic number), respectively.

^a^ The formulae used in these invariants, are simplified forms of general equations given that the vector $\;\bar{y}$ is constituted of the coordinates of the origin. For example, in the case of the Euclidean norm (N_2_), the general formula is: ${||\bar{x}||}_{2}=\sqrt{\sum_{i=1}^{n}{\left(x_{i}-y_{i}\right)}^{2}+{\left(x_{j}-y_{j}\right)}^{2}+{\left(x_{z}-y_{z}\right)}^{2}}$. However given that $\;\bar{y}$ = (0, 0, 0), this formula reduces to ${||\bar{x}||}_{2}=\sqrt{\sum_{i=1}^{n}{\left|x_{i}\right|}^{2}}$.

**Table 2:** Codification of the chain size, multiple bonds and their positions in the molecule.

Molecule	LOVIs Values						Total Invariants		
	C_1_	C_2_	C_3_	C_4_	C_5_	C_6_	N_1_	A	RA
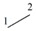	2	2	–	–	–	–	4	2	0
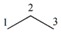	6	4	6	–	–	–	16	5.33	2
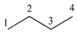	11.17	6.17	6.17	11.17	–	–	34.67	8.67	5
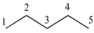	17.33	9.33	7.33	9.33	17.33	–	60.67	12.33	10
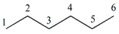	24.4	13.32	9.58	9.58	13.32	24.4	94.6	15.77	14.82
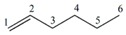	26.63	16.41	9.92	10.03	13.23	29.57	105.79	17.63	19.65
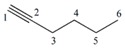	35.43	21.06	11.46	12.08	15.57	37.15	132.75	22.13	25.69
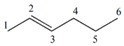	27.9	13.9	10.25	12.31	17.21	26.27	107.83	17.97	17.65
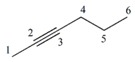	28.55	22.38	16.17	11.42	15.23	32.15	125.9	20.98	20.73
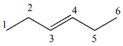	26.73	13.61	12.21	12.21	13.61	26.73	105.1	17.52	14.53
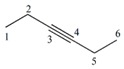	29.65	14.48	16	16	14.48	29.65	120.27	20.04	15.17
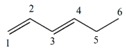	26.05	15.63	10.83	10.38	13.53	31.9	108.32	18.05	21.53
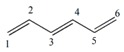	22.88	12.38	9	9	12.38	22.88	88.53	14.76	13.88

**Table 3:** Differentiation among chain isomers.

Molecule	LOVIs Values						Total Invariants		
	C_1_	C_2_	C_3_	C_4_	C_5_	C_6_	N_1_	A	RA
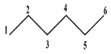	24.4	13.32	9.58	9.58	13.32	24.4	94.6	15.77	14.82
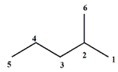	16.79	11.01	8.26	11.62	20.22	16.79	84.70	14.12	11.96
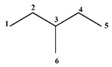	18.04	9.79	9.5	9.79	18.04	15	80.14	13.36	8.54
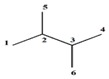	14.33	10.35	10.35	14.35	14.33	14.33	78	13	3.975
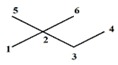	13.625	13.25	9.347	16.22	13.63	13.63	79.69	13.28	6.88

**Table 4:** Evaluation of some organic molecules with heteroatoms.

Molecule	Type of Atom in X	LOVIs Values				Total Invariants		
		C_1_	C_2_	C_3_	X	N_1_	A	RA
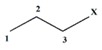	CH_3_	11.17	6.17	6.17	11.17	34.67	8.67	5.00
	NH_2_	11.37	6.87	6.64	12.54	37.41	9.35	5.90
	OH	11.69	7.44	7.02	13.81	39.95	9.99	6.79
	F	11.99	7.90	7.32	14.88	42.09	10.52	7.56

**Table 5:** Codification of cyclization, conjugation and aromaticity.

Molecule	LOVIs Values		Total Invariants		
	C_1_	C_2_	N_1_	A	RA
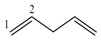	16.29	8.76	57.72	11.54	8.68
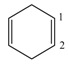	8.97	8.97	54.94	9.16	0.58
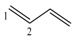	10.33	5.33	31.33	7.83	5
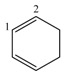	9.10	8.97	55.5	9.25	0.71
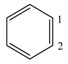	8.39	8.39	50.34	8.39	0
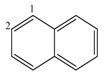	17.20	14.93	146.86	14.69	8.02
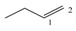	7.58	12.33	40.5	10.13	8.75
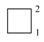	5.67	5.67	22.67	5.67	0
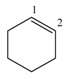	9.68	9.68	55.50	9.25	0.71
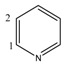	8.46	8.49	51.26	8.54	0.39
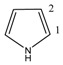	7.73	7.99	41.60	8.32	2.44

**Table 6:** Symmetry Index for some organic molecules.

Molecules	SI	Molecules	SI
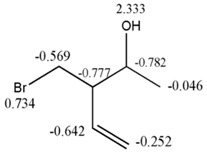	1.00	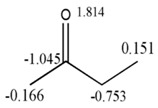	1.00
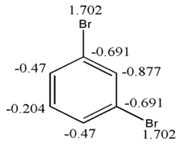	0.75	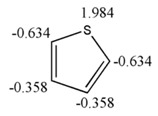	0.6554
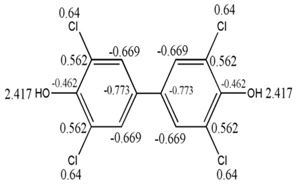	0.60	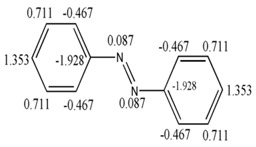	0.5870
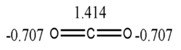	0.5793	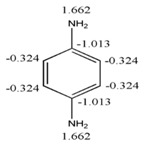	0.50
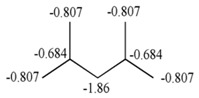	0.4911	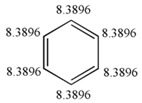	0.00

**Table 7:** Activation entropy and molecular interaction capacity for the dimerization of second order for unsaturated molecules in gaseous state.

Molecule	Ω (GDI)	ΔS^#^
Isobutylene	1.19988	−6
2-Isoprene	0.40035	−8
Ethylene	0	−12
1,3-Butadiene	0	−13
1,3-Pentadiene	−0.1948	−14
Propylene	−0.5501	−15
Cyclopentadiene	−2.1654	−26

**Table 8:** Accessibility to the chemical bond (Connectivity Indices and Graph Derivative Indices (GDIs)).

Name	Formula	R-GDI	RBA (*R*^2^)
**1**: ethane	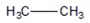	Infinite	1
**2**: propane	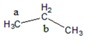	0.5	0.7071
**3**: methyl propane	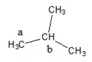	0.0833	0.5774
**4**: 2,2-dimetylpropane	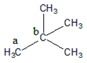	0.0278	0.5
**5**: *n*-butane	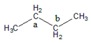	0.1111	0.5
**6**: methyl butane	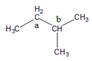	0.0357	0.4082
**7**: 2,2-dimethyl butane	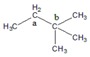	0.0154	0.3536
**8**: 2,3-dimethyl butane	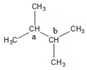	0.0156	0.3333
**9**: 2,2,3-trimethyl butane	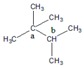	0.0079	0.2887
**10**: 2,2,3,3-tetramethyl butane	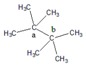	0.0044	0.25

**Table 9:** $C^{t}$ values for each type of carbon atoms.

Type of Atom	$H_{2}C=$	−CH=	$\dddot{-}\text{CH}\dddot{-}$	>C=	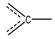	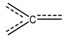
$C^{t}$	0.1364	−0.0371	−0.0778	−0.1211	−0.140	1.380

**Table 10:** Chemical Shift (δ) in ^17^O-NMR and GDI values.

No.	Compounds	E-State ^a^	[AN/In(IS)]BDf ^b^	δ (ppm) ^c^	δp (ppm) ^d^
1	CH_3_CHO	8.806	0.167	592.0	591.12
2	C_2_H_5_CHO	9.174	1.319	579.5	582.29
3	*i*-C_3_H_7_CHO	9.505	2.069	574.5	57 5.24
4	(CH_3_)_2_CO	9.444	3.250	569.0	564.41
5	CH_3_COC_2_H_5_	9.813	4.000	557.0	558.66
6	*i*-C_3_H_7_COCH_3_	10.144	4.417	557.0	554.57
7	(C_2_H_5_)_2_CO	10.181	5.000	547.0	550.41
8	*i*-C_3_H_7_COC_2_H_5_	10.512	5.833	543.5	542.41
9	(*i*-C_3_H_7_)CO	10.843	6.667	535.0	535.77

^a^ E-State Index; ^b^ Arithmetic mean of LOVIs from oxygen and carbon atoms in double bond; ^c^ Chemical shifts of the ^17^O-MNR; ^d^ Chemical shifts of the ^17^O calculated by Equation (22).

**Table 11:** Chemical Shift (δ) in ^17^O-NMR and GDI values.

No.	Compound	E-State ^a^	[AT/In(HT)]MD/f ^b^	δ (ppm)^ c^	δp (ppm) ^d^
1	Methoxymethane	4.20	42.58	−52.2	−53.12
2	Methoxyethane	4.54	35.74	−22.5	−22.64
3	2-Methoxypropane	4.75	28.80	−2	−1.56
4	2-Methoxy-2-methylpropane	4.94	25.15	8.5	9.36
5	Ethoxyethane	4.83	28.90	6.5	7.72
6	2-Ethoxypropane	5.04	21.96	28	28.75
7	2-Ethoxy-2-methylpropane	5.23	18.31	40.5	39.50
8	2-Isopropoxypropane	5.25	15.02	52.5	50.84
9	2-Isopropoxy-2-methylpropane	5.44	11.37	62.5	62.59
10	2-*t*-Butoxy-2-methylpropane	5.63	7.72	76	76.37

^a^ E-State Index; ^b^ LOVI of oxygen atoms; ^c^ Chemical shifts of the ^17^O-MNR; ^d^ Chemical shifts of the ^17^O calculated by Equation (23).

**Table 12:** Experimental resonance energy calculated by the GDI-based models.

Molecules	ERexp	ERcal (Ec. 24)	Res. ^a^	ERcal (Ec. 25)	Res. ^a^
Benzene	36	28.61	−7.39	35.17	0.83
Naphthalene	61	57.79	−3.21	57.5	3.5
Anthracene	83	86.97	3.97	80.77	2.23
Phenanthrene	91	86.25	−4.75	88.07	2.93
Styrene	38	39.47	1.47	29.54	8.46
Stilbene	74	74.81	0.81	70.95	3.05
Biphenyl	71	65.94	−5.06	69.54	1.46
Butadiene	3.5	12.29	8.79	4.57	−1.07
Fluorene	76	78.49	2.49	83.19	−7.19
3,5-Triphenylbenzene	149	140.6	−8.4	147.61	1.39
Toluene	35	33.14	−1.86	38.01	−3.01
*O*-Xylene	35	36.95	1.95	41.5	−6.5
Diphenylmethane	67	65.97	−1.03	71.6	−4.6
Naphthacene	110	116.15	6.15	111.58	−1.58
Chrysene	116.5	115.26	−1.24	115.32	1.18
Pyrene	108.9	107.11	−1.79	108.05	−0.05
Perylene	126.3	135.39	9.09	126.75	−0.45

^a^ Residual (ERexp–Ercal).
